# Transcriptome analysis provides insights into the cell wall and aluminum toxicity related to rusty root syndrome of *Panax ginseng*

**DOI:** 10.3389/fpls.2023.1142211

**Published:** 2023-06-13

**Authors:** Aizi Tong, Wei Liu, Haijiao Wang, Xiaoliang Liu, Guangqing Xia, Junyi Zhu

**Affiliations:** ^1^ Key Laboratory of Evaluation and Application of Changbai Mountain Biological Germplasm Resources of Jilin Province, College of Life Science, Tonghua Normal University, Tonghua, China; ^2^ College of Life Science, Changchun Normal University, Changchun, China

**Keywords:** *Panax ginseng*, rusty root, transcriptome, cell wall, aluminum tolerance

## Abstract

Rusty root syndrome is a common and serious disease in the process of *Panax ginseng* cultivation. This disease greatly decreases the production and quality of *P. ginseng* and causes a severe threat to the healthy development of the ginseng industry. However, its pathogenic mechanism remains unclear. In this study, Illumina high-throughput sequencing (RNA-seq) technology was used for comparative transcriptome analysis of healthy and rusty root-affected ginseng. The roots of rusty ginseng showed 672 upregulated genes and 526 downregulated genes compared with the healthy ginseng roots. There were significant differences in the expression of genes involved in the biosynthesis of secondary metabolites, plant hormone signal transduction, and plant–pathogen interaction. Further analysis showed that the cell wall synthesis and modification of ginseng has a strong response to rusty root syndrome. Furthermore, the rusty ginseng increased aluminum tolerance by inhibiting Al entering cells through external chelating Al and cell wall-binding Al. The present study establishes a molecular model of the ginseng response to rusty roots. Our findings provide new insights into the occurrence of rusty root syndrome, which will reveal the underlying molecular mechanisms of ginseng response to this disease.

## 1 Introduction

Ginseng (*Panax ginseng* C. A. Meyer), which commonly refers to the dried root and rhizome of *P. ginseng*, is known worldwide for its potent pharmacological activities, including its antiaging, antidiabetic, immunomodulatory, anticancer, and neuromodulatory properties (Kiefer and Pantuso, 2003; Zheng et al., 2012; Yang et al., 2014; Wang et al., 2016; Zheng et al., 2018). As a precious and important traditional Chinese medicine, ginseng has been cultivated for more than 4,000 years. Currently, the main cultivation areas of ginseng are in China and South Korea; it is also grown in Japan, North Korea, Canada, and the United States. The northeastern region is the most important ginseng production area in China. In addition, the phylogeny and population genetics of ginseng suggest that Fusong town in Northeast China might be the domestication center of Asian ginseng, as the variety grown there is closest to wild ginseng (Li et al., 2015). As a perennial medicinal plant, the active ingredients of ginseng will accumulate over time. Ginseng takes 4–6 years of consecutive cultivation to obtain high-quality ginseng with good medicinal properties. However, after 4 years of continuous planting, soil-borne diseases increased, resulting in significantly reduced yield and quality (Kim et al., 2017; Farh et al., 2018).

The rusty root is one of the most destructive root diseases of ginseng, characterized by small or large reddish-brown spots on the surface of the ginseng roots. This disease has attracted considerable attention due to its significant yield decline of between 20% and 30% per year and great economic losses (Garbeva et al., 2004). Rusty root-affected ginseng has less fibrous roots and reddish-brown to orange-brown irregular discolored lesions on the surface of the ginseng tap or lateral roots. In seriously diseased fields, sometimes even the whole root is covered with reddish-brown spots. Often, superficial lesions are easily scraped off, revealing the white healthy tissue inside. Presently, the cause of rusty root syndrome is still controversial. Some studies have shown that rusty root was closely related to the rhizosphere soil conditions (such as higher moisture, nitrate concentrations, and active Al species) and are considered a non-infectious physiological disease (Liu et al., 2014). Furthermore, microbes also play a key role in this disease, as suggested in other studies that ginseng root could be infected by putative pathogens, including bacteria (Choi et al., 2005; Lee et al., 2011), fungi (Rahman and Punja, 2005; Reeleder et al., 2006; Guan et al., 2014; Lu et al., 2015; Lu et al., 2020), or a combination of both (Ellouze et al., 2014; Liu et al., 2019), and showed rust symptom after inoculation of the isolates.

In this study, high-throughput sequencing was employed to systematically characterize changes in the healthy and rusty ginseng transcriptome. The results showed that the response of ginseng to the disease involved hormones (BR), Ca^2+^, HR, and cell wall-related signals. Our findings provide not only a new understanding of the origin of this disease but also a theoretical basis for its prevention and treatment.

## 2 Materials and methods

### 2.1 Plant materials

*P. ginseng* samples were collected in Henglu, Ji’an City, Jilin Province (41°34′65″N, 125°81′65″E; 428 m). These were randomly selected from 100 m^2^ of a 4-year ginseng plantation, and three healthy (CK) and rusty root (RR) ginseng plants were collected from the selected area as three biological replicates. The rusty ginseng samples were covered with red rust-colored lesions throughout the root.

The collected ginseng root tissues were rinsed with RNase-free water and then drained with clean paper. The ginseng root tissue was cut into small pieces ≤0.5 cm^2^ and quickly placed into the pre-cooled RNase-free threaded cryotube with written numbers. The samples were immediately frozen in liquid nitrogen for 1 h and then transferred to −80°C for long-term storage.

### 2.2 Library construction and sequencing

Total RNA Purification Kit, TRK1001 (LC Science, Houston, TX, USA), was used to extract the total RNA of six *P. ginseng* samples following the operation manual. RNA contamination and degradation were monitored on 1% agarose gels. The total RNA quantity and purity were measured using Bioanalyzer 2100 and RNA 1000 Nano LabChip Kit (Agilent, Santa Clara, CA, USA) with RIN number >7.0. Poly(A) RNA was purified from a total of 5 μg of RNA in two rounds using poly-T oligo-attached magnetic beads. After purification, the mRNA was fragmented into small pieces using divalent cations at elevated temperatures. The cleaved RNA fragments were then reverse transcribed according to the mRNA-seq sample preparation kit (Illumina, San Diego, CA, USA) to create the final cDNA library with an average insert size of 300 bp ( ± 50 bp) for the paired-end libraries. Then, we performed the paired-end sequencing on an Illumina HiSeq 4000 (LC Sciences, Houston, TX, USA) following the supplier’s recommended protocol.

### 2.3 Transcriptome analysis

We aligned sequencing reads to the Ginseng Genome Database (http://ginsengdb.snu.ac.kr/) reference genome using Hisat package v.2.0 (Kim et al., 2015). The Hisat software initially removed a part of the reads based on the quality information accompanying each read and then mapped the reads to the reference genome.

Mapped reads of each sample were assembled using StringTie (v.1.3.0) (Pertea et al., 2015). Then, all transcriptomes from samples were merged to reconstruct a comprehensive transcriptome using perl scripts. After the final transcriptome was generated, the expression levels of all transcripts were estimated using StringTie and edgeR. edgeR was used to perform the expression level of mRNAs by calculating FPKM (fragments per kilobase of transcript per million mapped reads). Differential expression analysis of the two groups was performed using the R package (v.3.2.5). The differentially expressed mRNAs and genes were selected using |log2(foldchange)| ≥ 1 (*p* < 0.05) as selection criteria.

### 2.4 Quantitative real-time PCR

The expression of differential expression genes was verified by quantitative real-time PCR (qRT-PCR) on a Real-Time PCR System (analytikjena-qTOWER2.2) using 2× SYBR^®^ Green Supermix (China). The specific primers of differentially expressed genes (DEGs) were designed by the online website (https://bioinfo.ut.ee/primer3-0.4.0/primer3/), and all of the primers used in this study are listed in **Table S2**. RNA was extracted from three biological replicates of rusty root-affected and healthy ginseng roots, and cDNA was synthesized using TUREscript 1st Strand cDNA Synthesis Kit (Aidlab). For each reaction system, 0.5 μl of the forward and reverse primers and 1 μl of the cDNA template were added. Conditions of qRT-PCR were as follows: 95°C (3 min), followed by 39 cycles of 95°C (10 s), 60°C (30 s), and 72°C (30 s). The 2^−ΔΔCt^ method was used to calculate the relative gene expression level (Arocho et al., 2006), which was log2 transformed and plotted against the corresponding means data from the RNA-seq and qRT-PCR.

### 2.5 Statistical analysis

In this study, all of the data are represented as the mean ± SD of the three technical replicates. Student’s *t*-test was used for statistical comparison between the CK and RR groups. Statistical analysis was performed with Microsoft Excel 2010. We considered *p* < 0.05 to be statistically significant.

## 3 Results

### 3.1 Transcriptome sequencing identified differentially expressed genes in rusty root

To gain a global overview of the rusty root transcript profile, healthy (CK) and rusty root-affected ginseng (RR) samples were sequenced by Illumina HiSeq 4000. Three biological replicates were designed in two groups. A total of six samples were obtained. In this study, a total of 267,199,488 raw reads were generated by sequencing CK *vs.* RR samples. After removing adaptor sequences and low-quality sequences, 49,741,788, 37,829,990, 37,235,008, 25,698,520, 46,584,096, and 40,707,226 clean reads were obtained (**Table 1**). These data were then mapped onto the Ginseng Genome Database (http://ginsengdb.snu.ac.kr/) reference genome using Hisat software, resulting in most of the valid reads being well-matched with the reference genome. It indicated that the transcriptome data were deemed suitable for the subsequent analysis.

**Table 1:** Summary of RNA-seq quality information.

Sample name	Raw reads	Clean reads	Clean bases	Clean ratio (%)	Q20 (%)	Q30 (%)	GC content (%)	Total mapped (%)	Exon (%)	Intron (%)	Intergenic (%)
CK1	51,971,074	49,741,788	7.46G	95.71	99.96	98.56	43	95.98	83.50	6.13	10.37
CK2	38,194,514	37,829,990	5.67G	99.05	99.94	98.13	43	95.57	83.44	6.04	10.52
CK3	51,223,996	37,235,008	5.59G	72.69	99.88	97.91	43	94.58	86.05	5.13	8.82
RR1	32,365,634	25,698,520	3.85G	79.40	99.10	96.06	44	94.48	87.27	4.39	8.34
RR2	51,297,154	46,584,096	6.99G	90.81	99.93	98.39	43	95.58	86.06	4.96	8.98
RR3	42,147,116	40,707,226	6.11G	96.58	99.94	98.17	43	95.03	85.71	5.24	9.05

Q20 (%) and Q30 (%): the percentage of bases with Phred values >20 and >30, respectively.

The expression levels of the genes from the ginseng transcriptomes were calculated and normalized to FPKM values. Through comparison with healthy ginseng samples, and using |log2(foldchange)| ≥ 1 (*p* < 0.05) as selection criteria, 1,198 genes (672 upregulated, 526 downregulated) with significant differential expression were identified in the diseased ginseng (**Table S1**). Compared with healthy ginseng, only external two to three layers of cells were affected and displayed a red to brown color in the rusty root-affected ginseng (Luo et al., 2022). In this study, we found that there was less differential expression of genes, which may be due to the high content of white ginseng root tissue (the unaffected root tissue).

**Figure 1:**
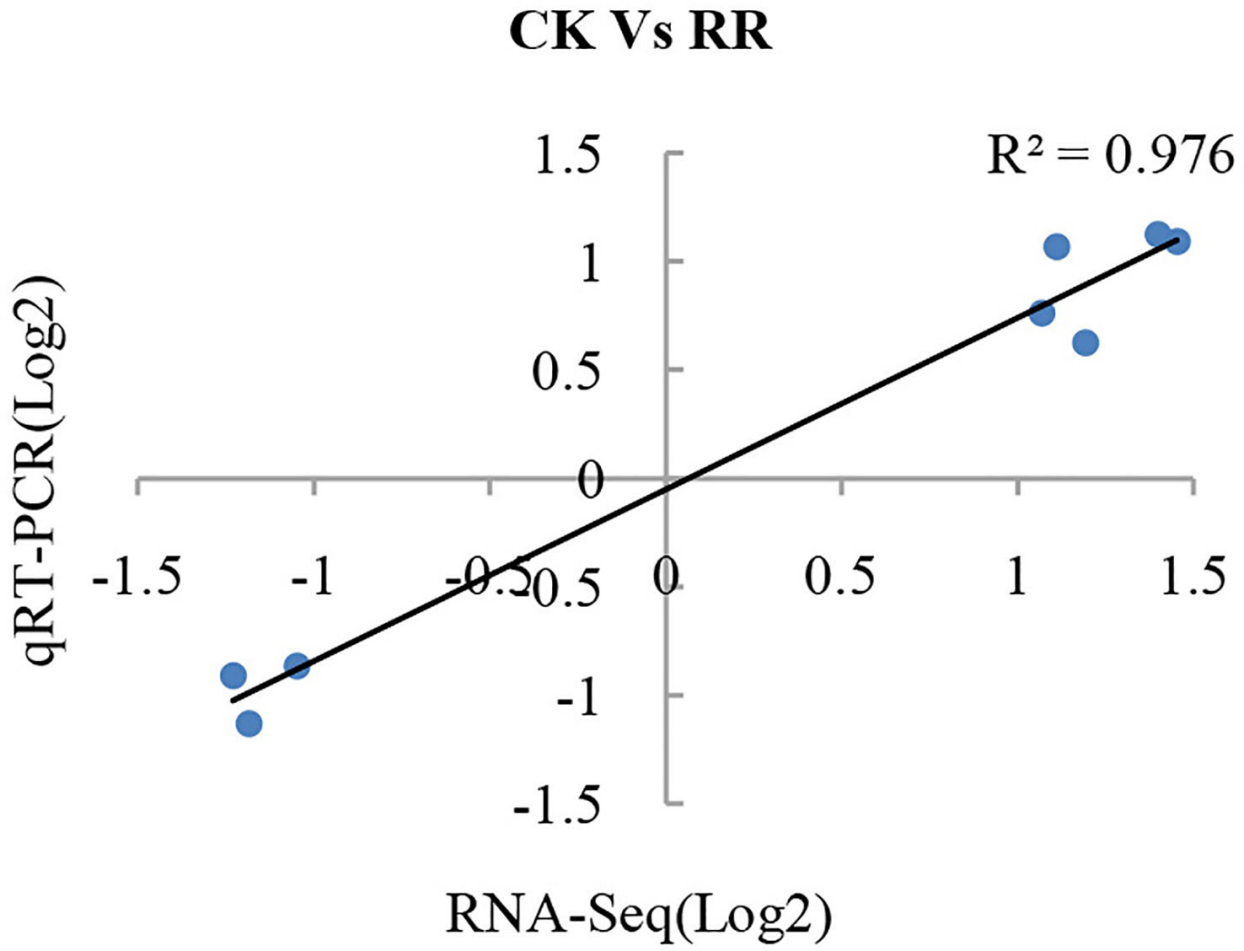
Validation of gene expression from RNA-seq by qRT-PCR analysis. Eight differentially expressed genes were randomly selected and used to examine the expression profiles by qRT-PCR analysis. The values from RNA-seq data were plotted against the corresponding qRT-PCR values and fitted into a linear regression. The R^2^ represents the correlation coefficient.

To validate the reliability of the RNA-seq data, eight DEGs were selected to investigate their transcriptional expression in the *P. ginseng* roots *via* qRT-PCR using gene-specific premiers (**Table S2**). To compare the expression data between RNA-seq and qRT-PCR, the relative expression level was transformed to log2(foldchange). The results showed a strong correlation between the RNA-seq and qRT-PCR data (R^2^ = 0.976; **Figure 1**), demonstrating the reliability of the RNA-seq expression profile in this study.

### 3.2 Functional classification of DEGs

To characterize the functions of all these DEGs, we performed gene ontology (GO) enrichment analysis to classify all the DEGs into 107 biological processes (BP), 18 cellular components (CC), and 52 molecular functions (MF) depending on *p*-value < 0.05 (**Table S3**). The DEGs in GO-enriched categories of the biological processes were mainly involved in the oxidation–reduction process, the ethylene-activated signaling pathway, the response to cadmium ion, and cell wall organization. In the cellular component, most of the DEGs were significantly mapped to the extracellular region, the membrane, the plasmodesma, and the cell wall. According to molecular function, the DEGs were mainly associated with transcription factor activity and oxidoreductase activity (**Table 2**). GO enrichment results showed DEGs with functions related to binding activity, oxidoreductase activity, and transferase activity after infection with the rusty root of ginseng. These genes primarily participated in biological processes such as gene function regulation, plant hormone regulation, defense response to microorganisms, response to abiotic stress, secondary metabolite biosynthetic process, and cell wall organization.

**Table 2:** GO enrichment analysis of up-and downregulated DEGs (*p*-value < 0.05).

GO function	GO ID	GO term	Upregulated DEGs	Downregulated DEGs	*p*-Value
Biological process	GO:0006355	Regulation of transcription, DNA-templated	61	34	0.00144
	GO:0006351	Transcription, DNA-templated	47	20	0.02437
	GO:0055114	Oxidation-reduction process	42	19	0.00000
	GO:0009873	Ethylene-activated signaling pathway	15	7	0.00001
	GO:0046686	Response to cadmium ion	14	8	0.01605
	GO:0071555	Cell wall organization	3	16	0.00011
	GO:0006636	Unsaturated fatty acid biosynthetic process	16	0	0.00000
	GO:0052696	Flavonoid glucuronidation	11	5	0.00003
	GO:0009813	Flavonoid biosynthetic process	11	5	0.00012
	GO:0006979	Response to oxidative stress	14	2	0.00284
	GO:0009414	Response to water deprivation	7	9	0.01419
	GO:0044550	Secondary metabolite biosynthetic process	9	6	0.00006
	GO:0042742	Defense response to bacterium	10	5	0.01615
	GO:0050832	Defense response to fungus	7	6	0.00308
	GO:0009611	Response to wounding	5	8	0.01348
	GO:0009860	Pollen tube growth	6	5	0.00201
	GO:0009753	Response to jasmonic acid	7	3	0.00291
Cellular component	GO:0005576	Extracellular region	29	30	0.00002
	GO:0016020	Membrane	33	24	0.02183
	GO:0009506	Plasmodesma	22	20	0.03007
	GO:0005618	Cell wall	14	18	0.00010
	GO:0005622	Intracellular	14	5	0.03808
	GO:0048046	Apoplast	8	10	0.01545
	GO:0043231	Intracellular membrane-bounded organelle	11	6	0.00505
	GO:0009505	Plant-type cell wall	9	7	0.00300
	GO:0046658	Anchored component of plasma membrane	5	6	0.02119
Molecular function	GO:0003677	DNA binding	54	32	0.00217
	GO:0003700	Transcription factor activity, sequence-specific DNA binding	52	32	0.00005
	GO:0043565	Sequence-specific DNA binding	17	10	0.00273
	GO:0016709	Oxidoreductase activity, acting on paired donors, with incorporation or reduction of molecular oxygen, NAD(P)H as one donor, and incorporation of one atom of oxygen	12	6	0.00000
	GO:0016491	Oxidoreductase activity	17	1	0.00724
	GO:0016717	Oxidoreductase activity, acting on paired donors, with oxidation of a pair of donors resulting in the reduction of molecular oxygen to two molecules of water	16	0	0.00000
	GO:0080043	Quercetin 3-*O*-glucosyltransferase activity	8	4	0.00076
	GO:0080044	Quercetin 7-*O*-glucosyltransferase activity	8	4	0.00076
	GO:0004553	Hydrolase activity, hydrolyzing *O*-glycosyl compounds	3	8	0.01282

GO, gene ontology; DEGs, differentially expressed genes.

**Figure 2:**
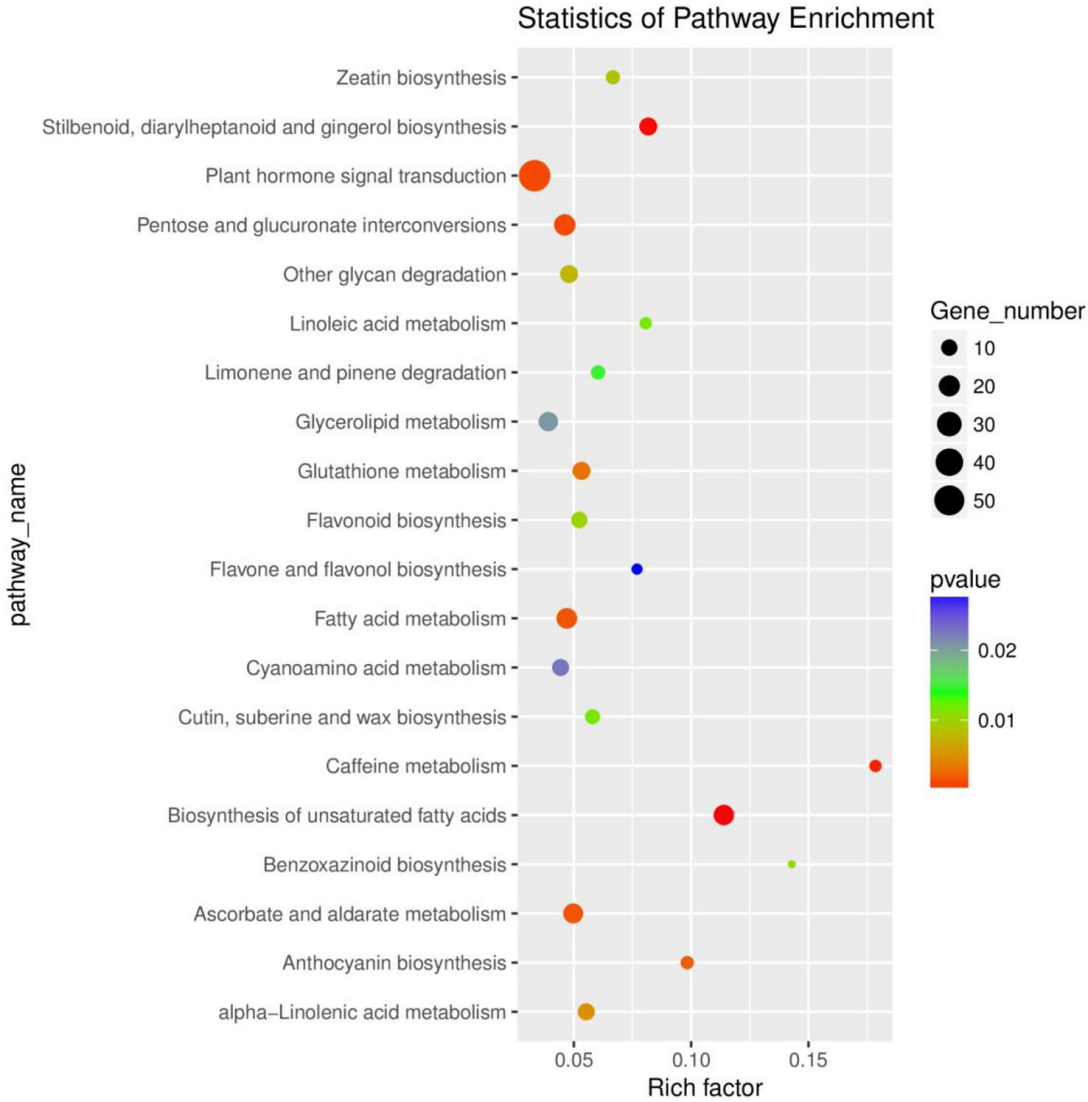
Functional classification of the DEGs *via* KEGG analysis. The top 20 enriched KEGG pathways are indicated, with dot size representing the ratio of selected genes to total genes in the pathway and dot color illustrating adjusted *p*-values. The ordinate shows the name of the KEGG terms, and the abscissa shows the ratio of the number of differentially expressed genes annotated to the pathway and the total of genes annotated to the pathway. Gene number and *p*-value are shown on the right. DEGs, differentially expressed genes; KEGG, Kyoto Encyclopedia of Genes and Genomes.

In addition, we performed the Kyoto Encyclopedia of Genes and Genomes (KEGG) enrichment analysis to identify the metabolic pathways involved in the responses of rusty roots in *P. ginseng*. Based on the rich factors and *p*-values, the DEGs were significantly enriched in 22 main pathways. Among these, plant hormone signal transduction, pentose and glucuronate interconversions, fatty acid metabolism, biosynthesis of unsaturated fatty acids, and ascorbate and aldarate metabolism were significantly enriched of the DEGs (**Figure 2**; **Table S4**). The enrichment analysis results of the GO and KEGG showed the responses of ginseng to rusty root syndrome. Compared with those in healthy ginseng, many DEGs in rusty root-affected ginseng were involved in plant hormone signal transduction, plant–pathogen interaction, and synthesis of cellulose and lignin.

According to the analysis, these pathways explained the possible roles of these DEGs in the occurrence of ginseng rusty roots. In the “biosynthesis of unsaturated fatty acids” pathway, most DEGs were annotated as omega-6 fatty acid desaturase/acyl-lipid omega-6 desaturase (Delta-12 desaturase) [EC:1.14.19.6 1.14.19.22], which usually played an active role in resisting environmental stresses such as cold, salt, and high temperature (Zhang et al., 2005; Wang et al., 2014), but their expression was mostly (17 of 18) promoted in the root of rusty ginseng (**Table S4**). The expression trend of these genes in diseased ginseng was contrary to the study of Gong et al. (2020).

### 3.3 Response of hormone-related genes to rusty root syndrome of ginseng

**Figure 3:**
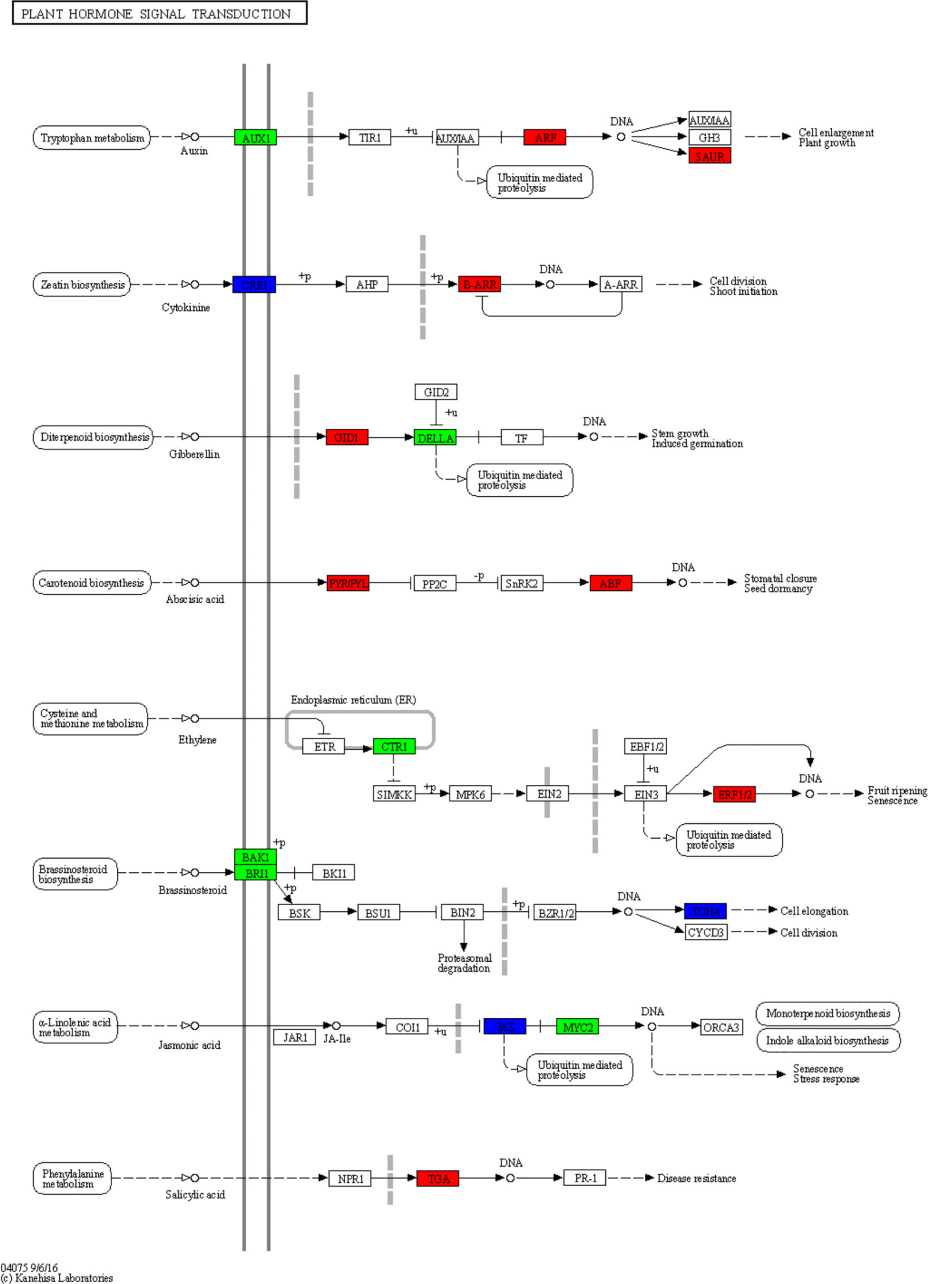
Overview of plant hormone signal transduction pathway. This diagram represents the signaling pathway of eight plant hormones in *Panax ginseng*. The different colors of the frame indicate the different gene transcription in RR compared with CK (red frame, upregulation; blue frame, downregulation).

**Figure 4:**
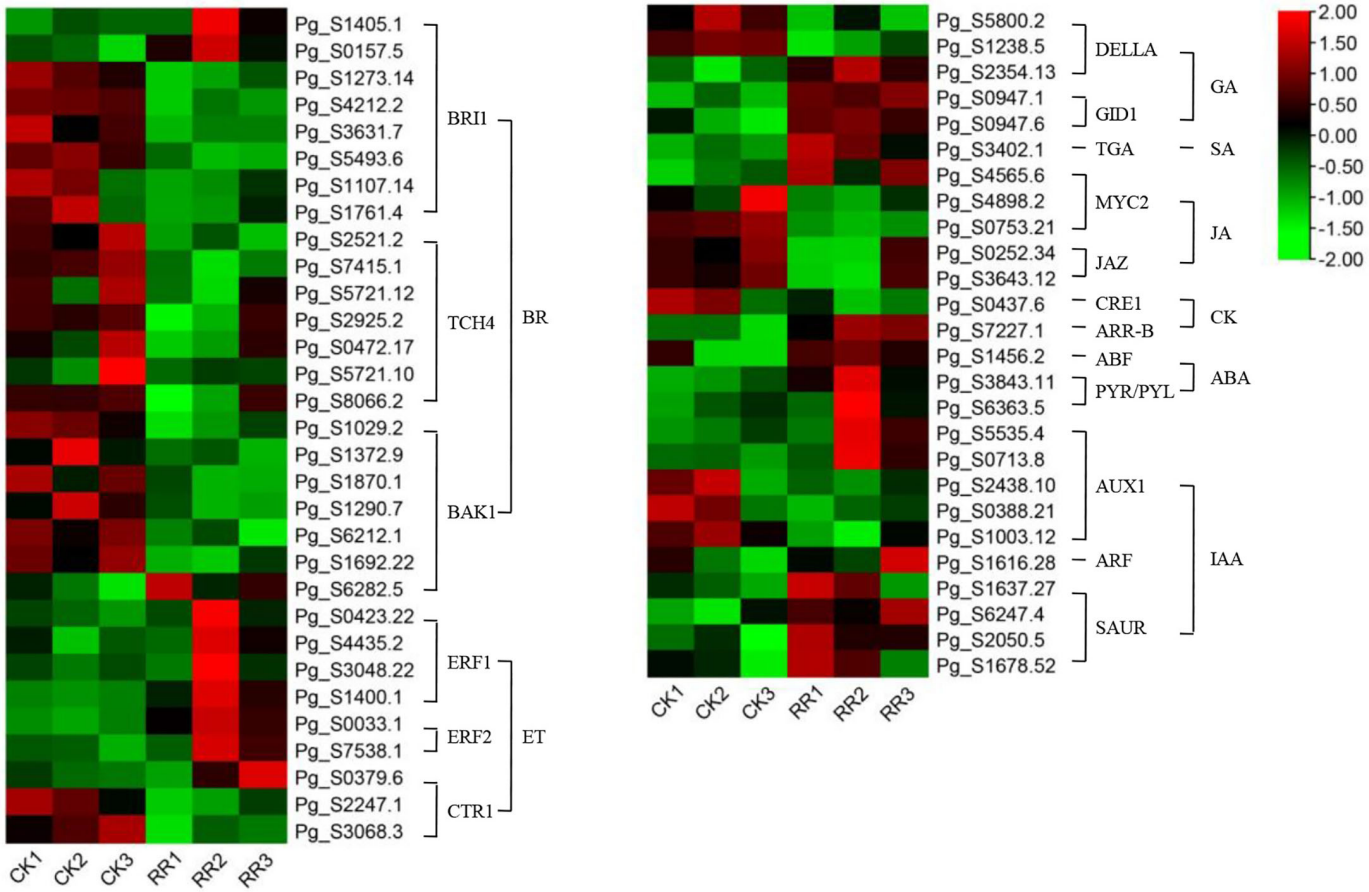
DEGs related to plant hormone signal transduction. The heatmap was constructed according to the expression level of these functional DEGs, and its log2-fold expression limit was 2 (red) to −2 (green). DEGs, differentially expressed genes.

Plant hormones are secondary metabolites that regulate plant growth and development at extremely low concentrations. Therefore, we further examine the transcription level of hormone-related genes. The analysis detected 84 DEGs involved in the brassinosteroid (BR), jasmonic acid (JA), auxin (IAA), ethylene (ET), gibberellin (GA), abscisic acid (ABA), cytokine (CK), and salicylic acid (SA) biosynthesis, metabolism, and signal transduction in rusty ginseng (**Figures 3**; **Table S5**). It shows that plant hormones play an important role in the response of ginseng to rusty root disease, especially BRs. Among these DEGs, 19 of the 22 BR metabolism and signaling pathway genes in rusty roots were significantly downregulated compared with healthy ginseng roots (**Figure 4**). All seven TCH4 (xyloglucan: xyloglucosyl transferase), which encode xyloglucan endotransglycosylase (XET), were downregulated in the roots of diseased ginseng (**Figure 4**). Furthermore, 15 DEGs (nine upregulated/six downregulated) were enriched in rusty root syndrome of ginseng compared with healthy ginseng (**Table S5**). These were primarily involved in JA metabolism and signaling pathways, including the phospholipase A1, lipoxygenase, 12-oxophytodienoic acid reductase, transcription factor MYC2, and jasmonate ZIM domain-containing protein (**Figure 4**; **Table S5**). Nine of the 14 IAA-related genes were upregulated in diseased ginseng, such as those encoding indole-3-pyruvate monooxygenase, *N*-hydroxythioamide *S*-beta-glucosyltransferase, auxin influx carrier (AUX1 LAX family), auxin response factor, and SAUR family protein (**Figure 4**; **Table S5**). Nine of the 12 ET-related genes were significantly upregulated in rusty roots, including those encoding aminocyclopropanecarboxylate oxidases, serine/threonine-protein kinase CTR1, ethylene-responsive transcription factor 1, and ethylene-responsive transcription factor 2 (**Figure 4**; **Table S5**). Furthermore, nine GA-related DEGs (seven upregulated/two downregulated), six ABA-related DEGs (three upregulated/three downregulated), five cytokine-related DEGs (four upregulated/one downregulated), and one SA-related DEG (upregulated) were enriched in diseased ginseng compared with healthy ginseng (**Figure 4**; **Table S5**).

### 3.4 DEGs involved in the “plant–pathogen interaction” pathway

**Figure 5:**
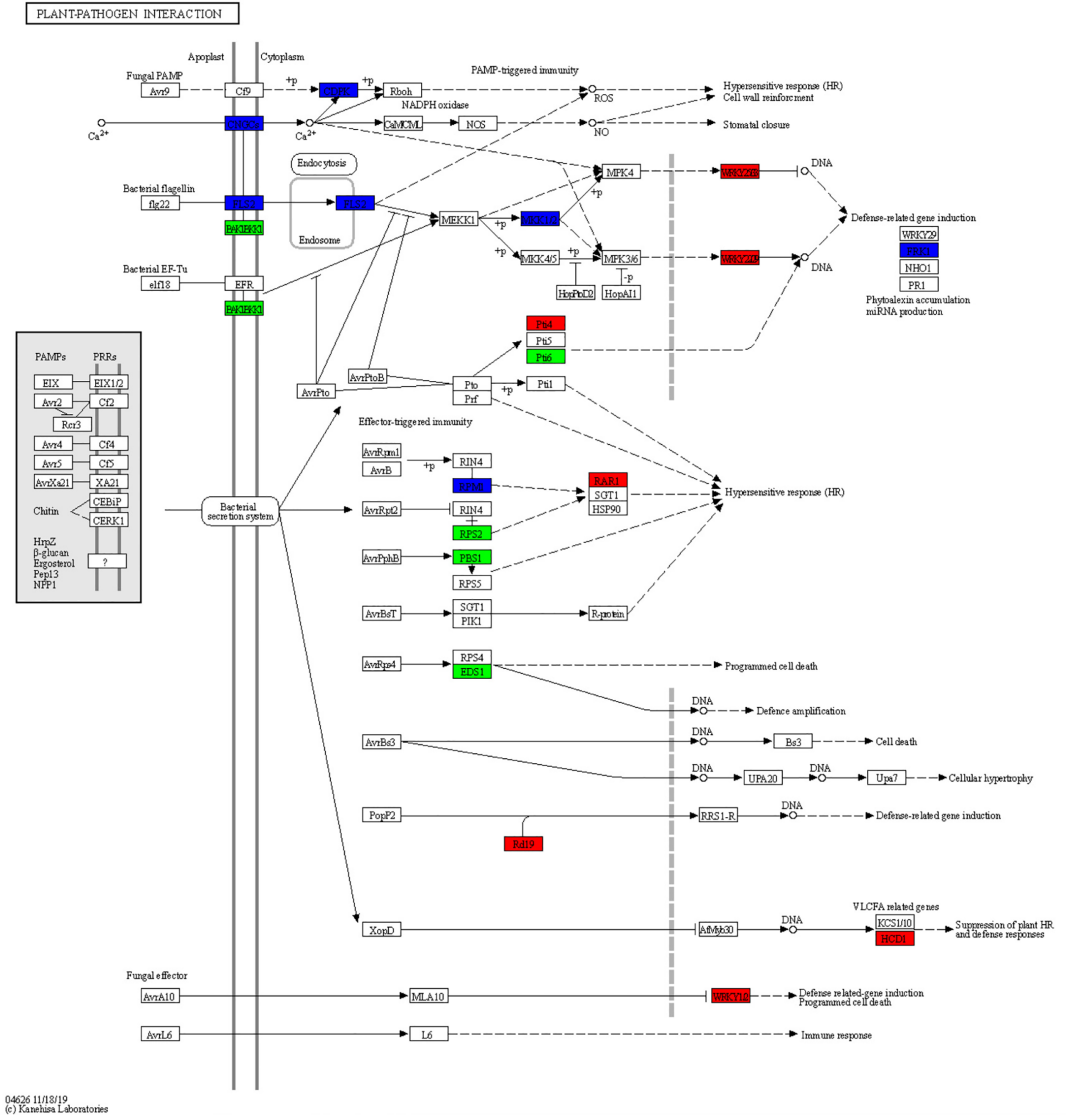
Overview of the plant–pathogen interaction pathway. The different colors of the frame indicate the different gene transcription in RR compared with CK (red frame, upregulation; blue frame, downregulation).

**Figure 6:**
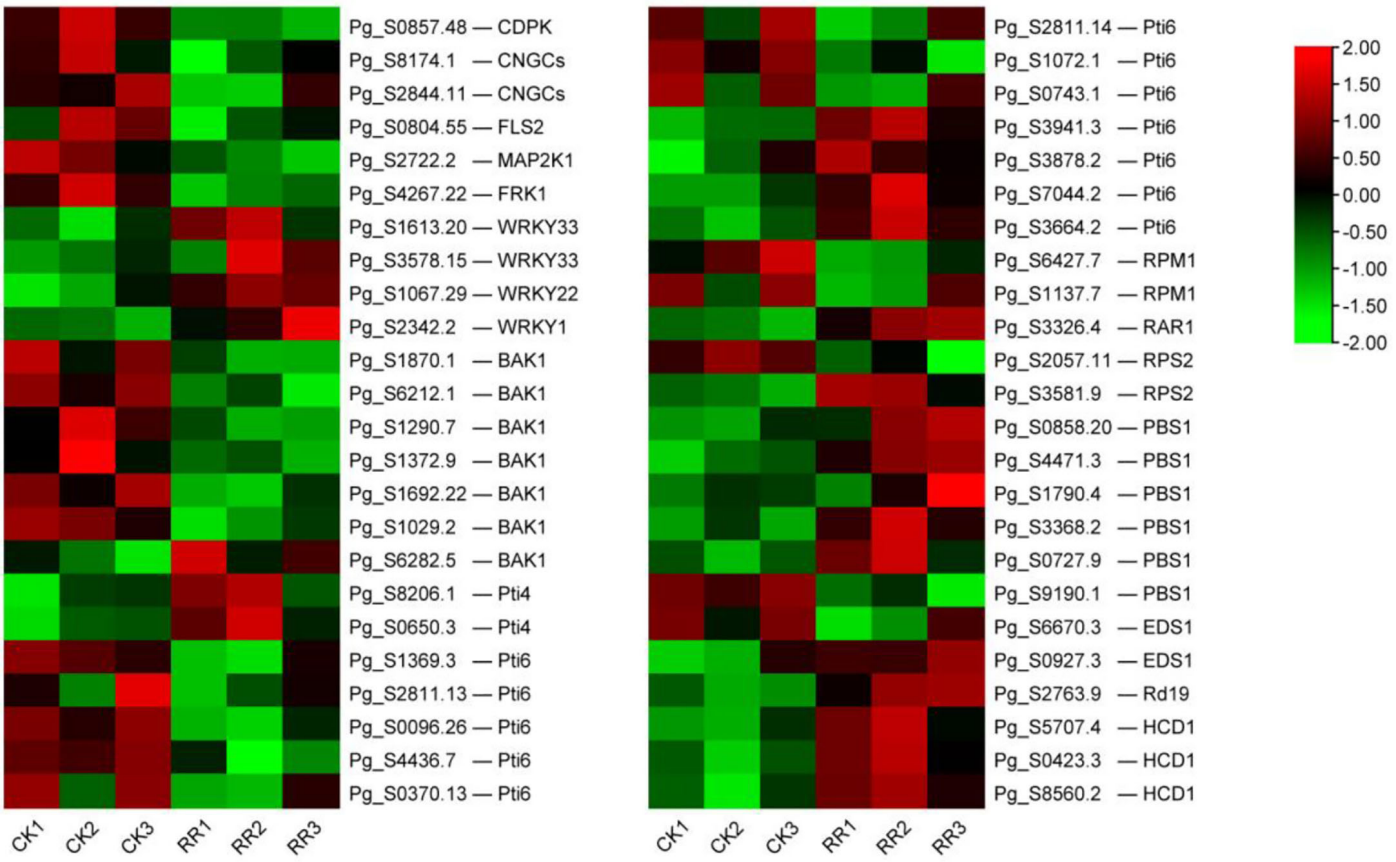
DEGs related to plant–pathogen interaction. The heatmap was constructed according to the expression level of these functional DEGs, and its log2-fold expression limit was 2 (red) to −2 (green). DEGs, differentially expressed genes.

There were 48 DEGs involved in the “plant–pathogen interaction” pathway (**Table S1**). It is speculated that these genes may play a key role in the response of ginseng to rusty roots. Compared with healthy ginseng, downregulation of Ca^2+^-dependent protein kinases (CDPKs) and cyclic nucleotide-gated channels (CNGCs), which affected Ca^2+^ flow and usually led to hypersensitive response (HR), were detected in diseased ginseng. Although FLS2 was downregulated, its downstream induced some transcription factors (TFs) responsible for inducing defense-related genes in rusty ginseng, such as WRKYs. In addition, several genes associated with effector-triggered immunity, including Pti4, Pti6, RPM1, RAR1, RPS2, PBS1, EDS1, Rd19, HCD1, and WRKY1/2, were distinctly differential expressed in rusty roots of ginseng (**Figures 5**, **6**).

CNGCs, which participate in the plant autoimmune cascade of early hypersensitivity, are one of the important Ca^2+^-conducting channels (Wang et al., 2022). CDPKs are usually involved in Ca^2+^ signaling cascades (Boudsocq and Sheen, 2013). As a secondary massager, Ca^2+^ plays a crucial role in activating the downstream defense reaction. The downregulation of CDPKs and CNGCs may be related to HR. FLS2, a pattern recognition receptor (PRR), can trigger innate immunity in plants. The downregulation of the above genes may indicate that part of PAMP-triggered immunity (PTI) is inhibited in rusty roots. Overall, the immune system of rusty root-affected was inhibited, thus affecting the HR and cell wall reinforcement, being sensitive to external stress, and reducing the survival rate of ginseng.

## 4 Discussion

### 4.1 The rusty root syndrome of ginseng is related to cell wall biosynthesis and modification

**Figure 7:**
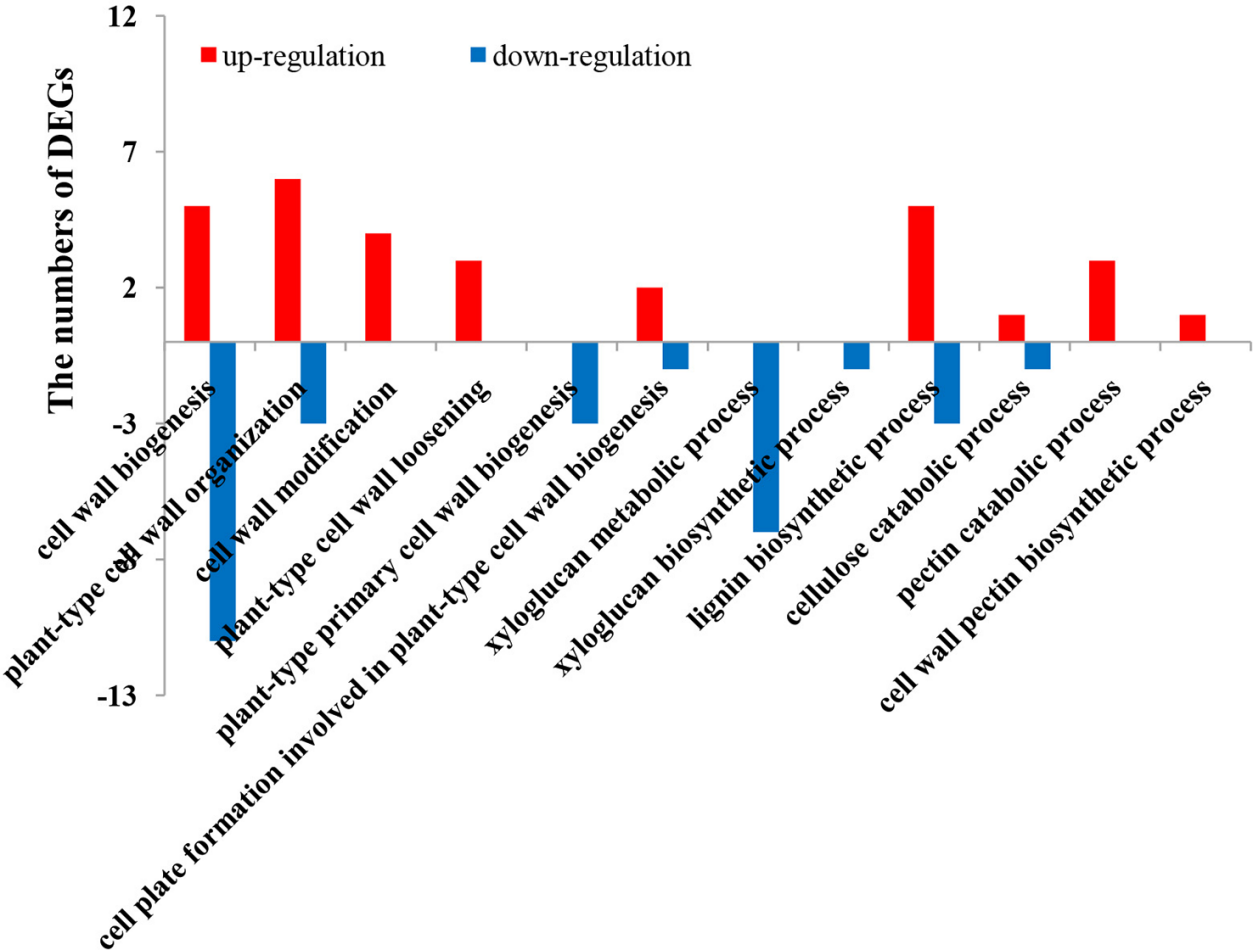
Statistics of GO terms related to the cell wall. GO, gene ontology.

**Figure 8:**
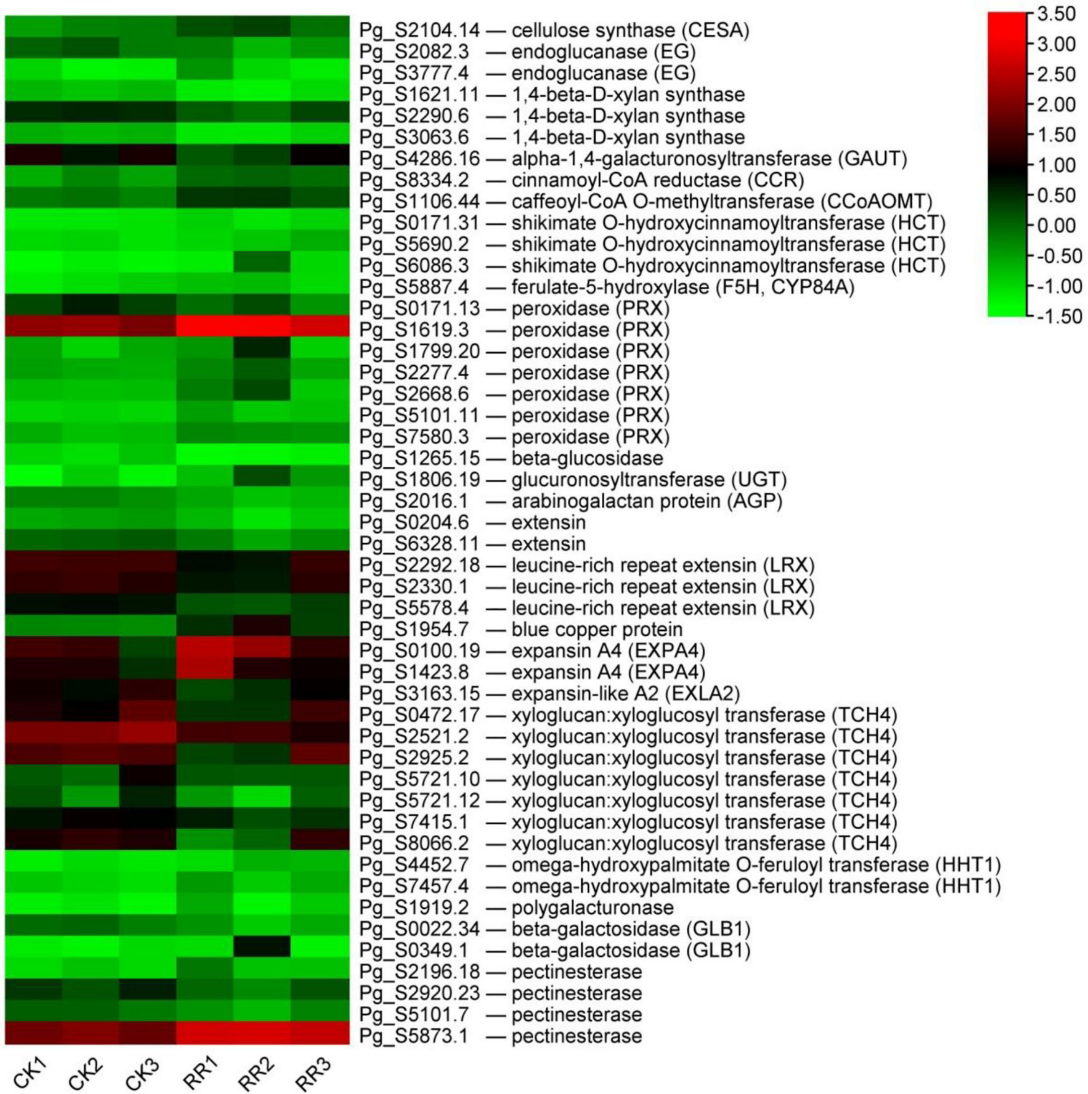
DEGs related to the cell walls. The heatmap was constructed according to the expression level of these functional DEGs, and its log2-fold expression limit was 2 (red) to −2 (green). DEGs, differentially expressed genes.

In addition to maintaining the cell structure, the cell wall also directly affects the response to environmental stresses, acting as a physical barrier (Yan et al., 2021). To confirm the response of cell walls in ginseng roots to rusty root syndrome, the relevant GO terms are listed in **Figure 7**. The analysis results discovered that many genes related to cell walls were differentially expressed in rusty ginseng, indicating that cell walls played a key role in the response of ginseng to rusty roots. There are two typical cell walls in plants: primary cell wall (PCW) and secondary cell wall (SCW). Their biosynthetic components and cellular locations are clearly distinguished. Each plant cell is surrounded by relatively thin and extensible PCW, which is mainly composed of three polysaccharide-derived polymers: cellulose, hemicelluloses, and pectin. SCW mainly includes cellulose, hemicelluloses, and lignin biopolymers. Cellulose in plants is synthesized by Cellulose Synthase Complex (CSC) containing at least three different cellulose synthases (CESAs) and also requires endo-1,4-beta-d-glucanase (EG) activity (Vain et al., 2014). In diseased ginseng, we found that one cellulose synthase (UDP-forming) gene was upregulated, and two EG genes were differential expression (**Figure 8**). Xylans are a diverse family of hemicellulosic polysaccharides found in abundance within the cell walls of nearly all flowering plants (Smith et al., 2022). Two transcripts of xylan synthase had inhibited expression in the rusty ginseng root (**Figure 8**). Pectin is composed of three polysaccharides, homogalacturonan (HG), rhamnogalacturonan I (RG-I), and rhamnogalacturonan II (RG-II). The most abundant pectic polysaccharide is HG, which is synthesized by GAUT gene family of α-1,4-galacturonosyltransferases (Mohnen et al., 2012). The occurrence of rusty root disease inhibits the expression of GAUT gene in ginseng. The biosynthesis of lignin, the main component of SCW, involves the complex regulation of many enzymes, such as cinnamoyl CoA reductase (CCR), caffeoyl-CoA *O*-methyltransferase (CCoAOMT), shikimate *O*-hydroxycinnamoyltransferase (HCT), and ferulate-5-hydroxylase (F5H, CYP84A) (Vanholme et al., 2010). The gene encoding CCR is a key gene for lignin biosynthesis, which is induced to express under stress conditions (Srivastava et al., 2015). CCoAOMT is an enzyme involved in monolignol synthesis that affects the efficiency of lignification and lignin composition (Rakoczy et al., 2018). Downregulation of HCT gene, an essential enzyme of the phenylpropanoid metabolism, reduces lignin content in alfalfa (*Medicago sativa* L.) (Bhattarai et al., 2018). F5H is a cytochrome P450-dependent monooxygenase, which is the enzyme responsible for the last hydroxylation of the syringyl-type lignin precursors (Franke et al., 2000). All DEGs encoding these four lignin synthetases are upregulated (**Figure 8**). Class III peroxidases (PRXs) promote lignin polymerization by oxidizing lignin monomers (monolignols) (Hoffmann et al., 2020; Zhu et al., 2021), although a beta-glucosidase, which is thought to play a key role in lignification by releasing the monolignol aglycones, inhibits the expression in the root of diseased ginseng (Dharmawardhana et al., 1995). Six of the seven DEGs related to lignin synthesis were upregulated in rusty roots (**Figure 8**). The transcriptional abundance of lignin synthesis-related transcripts increased, indicating the lignification process of rusty ginseng was enhanced. Lignin deposition is one of the responses to stress.

Arabinogalactan proteins (AGPs) and extensins are major members of the hydroxyproline-rich glycoprotein (HRGP) superfamily, which are unique components of plant cell walls (Tan et al., 2018). Glucuronosyltransferases are involved in the synthesis of the carbohydrate moieties of AGPs (Endo et al., 2013). AGPs in *Arabidopsis* positively regulate cell wall biosynthesis and root growth by regulating ABA signaling (Seifert et al., 2014; Hromadova et al., 2021). Extensins are involved in cell wall reinforcement in higher plants and defense against pathogen attacks (Castilleux et al., 2021). One AGP and five transcripts putatively coding for extension (including leucine-rich repeat extension, LRX) were all downregulated, while one glucuronosyltransferase was upregulated in the rusty root ginseng (**Figure 8**). LRXs modify cell wall expansion and also represent a connection between the cell wall and plasma membrane, perceiving extracellular signals and indirectly relaying this information to the cytoplasm (Herger et al., 2019). Additionally, blue copper protein, a gene putatively coding for a protein associated with the cell wall, was upregulated in rusty roots over fourfold (**Figure 8**). Cell wall modifications or alterations in cell wall structure are a common mechanism of defense against disease, and specific cell wall genes may contribute to plant defense. We found that there were differences in the expression profiles of cell wall-modifying genes. Expansins are plant cell wall-modifying proteins that have four subfamilies: α-expansin (EXPA), β-expansin (EXPB), expansin-like A (EXLA), and expansin-like B (EXLB) (Jin et al., 2020). As an important cell growth regulator, expansin also promoted interfascicular fiber cell elongation and cell wall thickness but did not alter the cellulose content in the cell wall (Li et al., 2017). XETs are most likely to modify the cell wall, which is the fundamental determinant of plant morphology (Xu et al., 1995). Two transcripts encoding EXPA were upregulated, while seven TCH4 and one EXLA were downregulated in the roots of diseased ginseng (**Figure 8**). Regulation of cell wall-modifying genes may underlie plant morphogenetic responses to the environment. BRs, a specific class of plant steroids, play a role in promoting growth involving cell division and elongation (Mandava, 1988; Hu et al., 2000; Sasse, 2003). Some BR-regulated genes have been proven to be related to cell elongation and cell wall organization (Goda et al., 2002). One of these genes is the TCH4 gene, which encodes the xyloglucan endotransglucosylase/hydrolase (XTH). It has been proposed that these specific XTH isoenzymes play a role in strengthening the lateral wall of root hairs and the cell walls of the root differentiation zone after the completion of cell expansion (Maris et al., 2009). The expression of the TCH4 gene in *Arabidopsis* is rapidly regulated by environmental variables, and changes in the expression level of the TCH4 gene directly lead to modifications in cell wall properties and structure (Xu et al., 1995). In our study, the mRNA levels of seven TCH4 genes were significantly reduced in the roots of rusty ginseng (**Figure 4**). It is revealed that the regulation of cell wall modifying enzyme genes is crucial to the regulation of plant morphogenetic response to the environment (Surgun and Burun, 2015). The reason for regulation is to change the properties of the cell walls so that plants can rapidly adapt to environmental conditions (Xu et al., 1995; Surgun and Burun, 2015). Our research results indicated that the occurrence of rusty root disease induces the cell wall modification of ginseng roots, which may also be one of the mechanisms for ginseng to adapt to stress. Omega-hydroxypalmitate *O*-feruloyl transferase (HHT1) could be involved in suberin biosynthesis. Suberin is a cell wall biopolymer with aliphatic and aromatic domains, which is synthesized in plant wound tissues to restrict water loss and pathogen infection (Molina et al., 2009). It was found that two HHT1 genes were induced to express in diseased ginseng (**Figure 8**). In addition, several cell wall-degrading enzymes (mainly polygalacturonase, beta-galactosidase, and pectinesterase), leading to cell wall loosening, were differential expressions (**Figure 8**). The modification in the cell wall may contribute greatly to the occurrence of rusty root syndrome.

### 4.2 The rusty root syndrome of ginseng is related to aluminum stress

**Figure 9:**
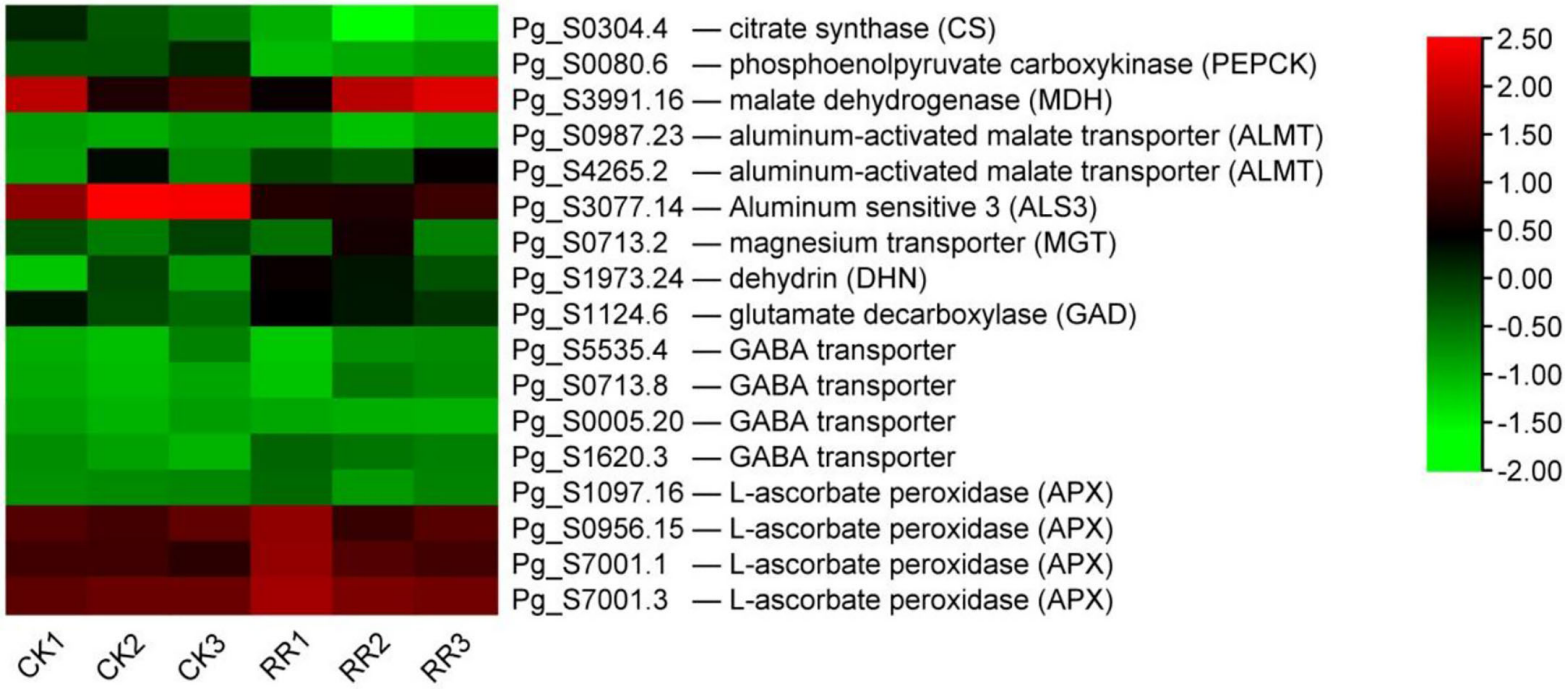
DEGs related to aluminum stress. The heatmap was constructed according to the expression level of these functional DEGs, and its log2-fold expression limit was 2 (red) to −2 (green). DEGs, differentially expressed genes.

Chinese scholars have reported that continuous cultivation of ginseng results in soil acidification, and the content of active Al in soil increases (Liu et al., 2014; You et al., 2015). Aluminum stress is believed to be related to the rusty root syndrome of ginseng (Zhou et al., 2017; Ma et al., 2021). Therefore, this study analyzed the expression of genes related to aluminum stress from the transcriptional level. Organic acid ions (including malate, citrate, and oxalate) secreted by plant roots can directly chelate external Al to prevent aluminum toxicity (Ma et al., 2001; Ryan et al., 2001). The expression of citrate synthetic enzyme CS (citrate synthase) and PEPCK (phosphoenolpyruvate carboxykinase, utilized in the dissimilation of malate/citrate) were decreased, while malate synthase MDH (malate dehydrogenase) was increased in rusty ginseng (**Figure 9**). Organic acids produced by plants are exuded outside the root to the rhizosphere through membrane transporters. The malate transporter ALMT (aluminum-activated malate transporter) and the citrate transporter MATE (multidrug and toxin extrusion) facilitate root malate and citrate exudation, respectively (Hoekenga et al., 2006). Transcription levels of two ALMTs were significantly upregulated in rusty ginseng roots (**Figure 9**). This study speculated that the synthesis and outward transportation of malate in ginseng roots were enhanced during the process of ginseng infection with rusty roots, indicating that malate played an important role in the rusty roots. In *Arabidopsis thaliana*, plant roots secrete both malate and citrate in response to Al stress, with malate being the major player in Al resistance (Liu et al., 2009). Accordingly, the increased malate synthesis and secretion might contribute significantly to the enhanced Al resistance in rusty ginseng, while the negative effect of reduced citrate synthesis on the Al resistance is negligible.

In addition to the organic acid exudation-based Al-resistance mechanism, another effective strategy for Al exclusion is the absorption of Al by plant cell wall polysaccharides. At a high Al concentration in the environment, barley may absorb ~85% of the peripheral Al into its root cell walls (Wang et al., 2006), and the giant alga Chara coralline may even absorb up to 99.9% of total Al into its cell walls (Taylor et al., 2000). The total aluminum content in the rusty ginseng was higher, and it mainly accumulated in the diseased root and its periderm (Zhou et al., 2017). The internal detoxification of plants utilizes the ALS (aluminum-sensitive) encoding tonoplast-localized ATP-binding cassette (ABC) transporter to chelate internal Al into the vacuoles (Larsen et al., 2007). The bacterial-type ABC transporter complex STAR1/ALS3 is responsible for the transport of UDP (uridine diphosphate)-glucose that can modify cell walls and therefore masks Al-binding sites (Larsen et al., 2005). The transcription level of ALS3 was inhibited in the rusty ginseng root (**Figure 9**). It indicated that the Al-binding sites of cell walls were exposed, while the internal detoxification of aluminum was weakened. The results of this study revealed that aluminum tolerance in rusty ginseng roots was mainly through external chelation aluminum and cell wall-binding aluminum to restrain aluminum into cells. Therefore, it is considered that the rusty root syndrome of ginseng is a spontaneous self-protection mechanism of ginseng under aluminum stress for a long time.

Some genes related to aluminum tolerance were induced to express in ginseng root infected with the rusty syndrome. It is reported that magnesium transporter (MGT) is one of the cellular targets of Al toxicity, and overexpression of MGT1 confers Al tolerance on the plant (Deng et al., 2006). Dehydrins (DHNs) are considered molecular protectors. MsDHN1 is involved in increasing the tolerance of alfalfa to Al stress (Lv et al., 2021). GABA has been reported to regulate the malate-transporting plasma membrane channel during Al stress in wheat (Ramesh et al., 2015; Ramesh et al., 2018). GABA synthetase glutamate decarboxylase (GAD) and four GABA transporters are upregulated (**Figure 9**). Ascorbate (AsA) and glutathione (GSH) are at the heart of the redox hub, and AsA–GSH system plays pivotal roles in Al tolerance. The changes in the levels and status of AsA and GSH were correlated with alterations in AsA–GSH cycle-allied enzymes, including ascorbate peroxidase (APX), dehydroascorbate reductase (DHAR), monodehydroascorbate reductase (MDHAR), and glutathione reductase (GR). Compared with healthy ginseng, the transcriptional levels of four APXs were upregulated, while the expression levels of DHAR, MDHAR, and GR had no significant changes (**Figure 9**). The above results indicate that aluminum resistance-related genes are induced to express, and aluminum resistance function is activated in the root of rusty ginseng.

Transcriptome analysis provided clues for the molecular mechanism of ginseng-infected rusty roots. In conclusion, the immune system of rusty ginseng was inhibited, and the lignification process and the cell wall were enhanced. The aluminum resistance-related genes were induced, and the synthesis and outward transportation of malate were enhanced in the rusty roots of ginseng. It was indicated that the rusty ginseng increased aluminum resistance, and aluminum resistance mainly inhibited aluminum from entering cells through external chelating aluminum and cell wall-binding aluminum. Therefore, it is considered that ginseng rusty root syndrome is the spontaneous self-protection mechanism of ginseng under long-term aluminum stress.

## Data availability statement

The datasets presented in this study can be found in online repositories. The names of the repository/repositories and accession number(s) can be found below: NCBI Sequence Read Archive (SRA) database (Accession Number: PRJNA941146).

## Author contributions

AT and WL contributed to the experimental design and manuscript writing. HW and XL contributed to the data analysis. GX and JZ contributed to the qRT-PCR verification experiment. All authors contributed to the article and approved the submitted version.
