# Supplementary material for: Transcriptome analysis provides insights into the cell wall and aluminum toxicity related to rusty root syndrome of Panax ginseng
Source: Front Plant Sci. 2023 Jun 13;14:1142211. doi: 10.3389/fpls.2023.1142211 (PMC10293891; doi:10.3389/fpls.2023.1142211)
Supplement: Supplementary file 2 [file Table_2.doc]

**Table S2 Primer sequences used in qRT-PCR**

| Genes | Forward primer (5’-3’) | Reverse primer (5’-3’) |
| --- | --- | --- |
| *Pg_S1993.2* | GTCCTTGATGTCTGCCATTG | ATATCCCTGCTTGACCTGTT |
| *Pg_S1029.2* | GCTAGGCTGTTACCAATGC | AGCACCACCACATCATCT |
| *Pg_S0857.48* | CAAGCGAAGCATTTGATGTATC | GAGGATTCTCACAACGAACTAA |
| *Pg_S0530.2* | GGCCTGCTGAGCTTATAGAT | CGGGAGTAGTGAGCATAGTG |
| *Pg_S0100.19* | TGATGATGAGCCGTAACTG | GGGTACAATGTTCCGAGAA |
| *Pg_S3402.1* | AACTTGAGCCGTTGACTG | TGATTCCAGCCTCTCCAT |
| *Pg_S2104.14* | ACGGTCAAGGGAACAATG | CCGCCACCTACTTCTTTC |
| *Pg_S1619.3* | TGCGAATGCGATGATAAAGATG | CACCACAGAAGACAACAACAAA |
| *Actin* | CTTGCACCAAGCAGCATGAA | CCGATCCAGACACTGTACTTCCTT |
